# Estimating Costs Associated with a Community Outbreak of Meningococcal Disease in a Colombian Caribbean City

**Published:** 2014-09

**Authors:** Hernando Pinzón-Redondo, Wilfrido Coronell-Rodriguez, Inés Díaz-Martinez, Ángel Guzmán-Corena, Dagna Constenla, Nelson Alvis-Guzmán

**Affiliations:** ^1^Centro de Investigación y Docencia, Hospital Infantil Napoleón Franco Pareja, Cartagena-Colombia; ^2^Grupo de Investigación en Economía de la Salud, Universidad de Cartagena, Cartagena-Colombia; ^3^Facultad de Medicina, Universidad de Cartagena, Cartagena-Colombia; ^4^International Vaccine Access Center (IVAC), Johns Hopkins Bloomberg School of Public Health, Baltimore, Maryland, USA

**Keywords:** Chemoprophylaxis, Cost, Invasive meningococcal disease, Outbreak

## Abstract

Meningococcal disease is a serious and potentially life-threatening infection that is caused by the bacterium *Neisseria meningitidis* (*N. meningitidis*), and it can cause meningitis, meningococcaemia outbreaks and epidemics. The disease is fatal in 9-12% of cases and with a death rate of up to 40% among patients with meningococcaemia. The objective of this study was to estimate the costs of a meningococcal outbreak that occurred in a Caribbean city of Colombia. We contacted experts involved in the outbreak and asked them specific questions about the diagnosis and treatment for meningococcal cases during the outbreak. Estimates of costs of the outbreak were also based on extensive review of medical records available during the outbreak. The costs associated with the outbreak were divided into the cost of the disease response phase and the cost of the disease surveillance phase. The costs associated with the outbreak control and surveillance were expressed in US$ (2011) as cost per 1,000 inhabitants. The average age of patients was 4.6 years (SD 3.5); 50% of the cases died; 50% of the cases were reported to have meningitis (3/6); 33% were diagnosed with meningococcaemia and myocarditis (2/6); 50% of the cases had bacteraemia (3/6); 66% of the cases had a culture specimen positive for *Neisseria meningitidis*; 5 of the 6 cases had RT-PCR positive for *N. meningitidis*. All *N. meningitidis* were serogroup B; 50 doses of ceftriaxone were administered as prophylaxis. Vaccine was not available at the time. The costs associated with control of the outbreak were estimated at US$ 0.8 per 1,000 inhabitants, disease surveillance at US$ 4.1 per 1,000 inhabitants, and healthcare costs at US$ 5.1 per 1,000 inhabitants. The costs associated with meningococcal outbreaks are substantial, and the outbreaks should be prevented. The mass chemoprophylaxis implemented helped control the outbreak.

## INTRODUCTION

Invasive meningococcal disease is a potentially life-threatening infection (1). About 500,000 cases of the disease occur worldwide each year (2), of which approximately 800-1,500 cases occur annually in the United States, resulting in a rate of 0.3-0.5/100,000 population (3,4). In developing countries, meningococcal disease causes 330,000 cases annually and results in 35,000 annual deaths. The estimated total numbers of deaths reported range from 350,000 to 600,000 annually, especially in infants below one year of age (5,6).

Meningococcal disease can result in outbreaks and epidemics (2). *N. meningitidis* can be classified into 13 serogroups based on their capsular polysaccharides. Serogroup B causes over 50% of cases in infants (<1 year of age) while serogroups C, Y, and W135 combined cause 75% of meningococcal disease in children aged 11 years and older (7). Vaccines for serogroup A, C, Y, and W135 are available but nowadays no vaccine is available for serogroup B.

We describe the outbreak of meningococcal disease in a Colombian Caribbean city with 1 month of active surveillance follow-up. In this outbreak, a total of 6 cases were detected, of which 3 resulted in deaths. While infants are most commonly affected by meningococcal disease, adolescents have the highest rates of mortality (1,2). Invasive meningococcal disease is caused by *Neisseria meningitidis* (NM). Serogroups B and C are the main causes of meningococcal disease in the Americas and in Europe. The meningococcus can be transmitted from human to human through direct contact with droplet of respiratory secretions (8). *N. meningitidis* causes a number of clinical conditions, including meningitis and bacteraemia (or meningococcaemia), which are the most severe manifestation of meningococcal infection; and much less common localized infection, such as pneumonia, pericarditis, endocarditis, supraglottitis, conjunctivitis, urethritis, and otitis media (3,9-15). The disease is fatal in 9-12% of cases, with a death rate of up to 40% in patients with meningococcaemia (16,17).

The costs associated with management of cases and outbreaks of meningococcal disease in Latin America and the Caribbean region are underreported and underestimated in the literature. The studies that estimate the costs of meningococcal disease are limited and provide information from Europe and other parts of the world (18).

We estimated the costs associated with an outbreak of serogroup B meningococcal disease in Colombia.

## MATERIALS AND METHODS

We describe an outbreak of serogroup B meningococcal disease (six cases) in the city of Cartagena de Indias in Colombia. Cartagena de Indias, located on the shores of the Caribbean Sea, is one of the most important tourist destinations in Latin America, with a population of nearly one million inhabitants.

The outbreak occurred from 21 February through 17 March 2012 and involved 6 children who lived in a low-income neighbourhood in the southeast of the city. The outbreak was investigated by the Napoleon Franco Pareja Children's Hospital (CH) and the District Health Department (DADIS, Spanish acronym for Departamento Administrativo Distrital de Salud). The CH is a tertiary academic hospital and is the largest paediatric hospital in the Colombian Caribbean region whereas the DADIS is a governmental agency that regulates and monitors disease outbreaks.

The costs were split in two phases: the disease response phase that includes costs associated with management of the disease and the disease surveillance phase cost that corresponds to monitoring of disease cases.

### Characteristics of patients

Twenty close contacts were found, and they were admitted to hospital (CH) for medical check-up. Close contacts were defined as patients who have meningococcal disease and may include: (i) household members (including dormitory room, barracks); (ii) childcare centre contacts, and (iii) persons directly exposed to the patient's oral secretions (e.g. by kissing, mouth-to-mouth resuscitation, endotracheal intubation, or endotracheal tube management) 10 days preceding hospitalization (7,19-22). We found 6 confirmed cases of invasive meningococcal disease, defined as an isolated case of *N. meningitidis* from a normally-sterile body site. A probable case was defined as *N. meningitidis* DNA detected by polymerase chain reaction without organism isolation in a suspected patient. A suspected case was defined as physician-reported fever, and any rash in a person was linked epidemiologically to a patient with a confirmed case (Table 1). The associated myocarditis has been considered in the literature as the cause of non-response to inotropic support and is the predominant cause of death in meningococcal disease (23). The results of the pathologic study consistent with meningitis and Waterhouse Friderichsen syndrome were regarded (24).

### Cost associated with the outbreak

We estimated the costs of the meningococcal outbreak by splitting the direct treatment costs and costs associated with outbreak control (18). The costs considered were personnel costs, measured in time and estimated as a fraction of the salary of each. An outbreak care team consisted of two paediatricians, two nurses, an epidemiologist, and two public health experts. We performed a microcosting analysis of 5 patients. One patient (patient C) was excluded from the analysis because the patient died before arriving at the health centre, and no data on treatment management were available.

Costs associated with mass chemoprophylaxis were based on the guidelines of the American Academy of Pediatrics. These guidelines recommend oral rifampin in 4 doses over 2 consecutive days or a single-dose intramuscular ceftriaxone injection for use as chemoprophylaxis against meningococcal disease among children (25).

**Table 1.** Patient, clinic, and laboratory characteristics involving confirmed, probable and suspect cases of meningococcal disease (N=6) based in the Colombian Caribbean City, February 2012

Case	A	B	C	D	E	F
Age	1.5 years	11 years	5 years	3 years	5 years	2 years
Gender	Male	Female	Male	Female	Female	Male
Onset of illness (date)	21/02/2012	22/02/2012	25/02/2012	28/02/2012	24/02/2012	28/02/2012
Hospital admission (date)	28/02/2012	25/02/2012	25/02/2012	28/02/2012	28/02/2012	02/03/2012
Hospital discharge (date)	28/02/2012	27/02/2012	25/02/2012	02/03/2012	02/03/2012	10/03/2012
Confirmed?	Confirmed	Probable	Suspect	Confirmed	Confirmed	Confirmed
Fever	Yes	Yes	No	Yes	Yes	Yes
Vomit	Yes	Yes	No	No	No	No
Myalgia	No	No	No	No	No	No
Drowsiness	Yes	Yes	Yes	No	No	No
Headache	No	Yes	No	No	No	Yes
Seizures	No	Yes	No	No	No	No
Purpuric rash	Yes	No	No	No	No	No
Leukocytes	Leukopenia	Leukocytosis	Normal	Normal	Normal	Normal
CSF	Normal	Altered	No	No	No	No
CSF culture	Negative	Negative	No	No	No	No
Bacteraemia	Yes	No	No	Yes	Yes	Yes
Meningitis	Yes (autopsy)	Yes	Yes (autopsy)	No	No	No
Meningococcaemia	Yes (autopsy)	No	Yes (autopsy)	No	No	No
Myocarditis	Yes (autopsy)	No (autopsy)	Yes (autopsy)	No	No	NO
ICU	Yes	Yes	No	No	No	No
Culture specimen	Blood	Blood	No (autopsy)	Blood	Blood	Blood
Result	Positive	Negative	Negative	Positive	Positive	Positive
Isolated germ	NM	No	No	NM	NM	NM
Serogroup	B	No	No	B	B	B
Conventional PCR (*crg*A) specimen	CSF	CSF	No	No	No	No
Result PCR (*crg*A)	Negative	Negative	No	No	No	No
Real-time PCR (RT-PCR) specimen	CSF	CSF	No	Blood	Blood	Blood
Result RT-PCR	Positive (NM Serogroup B)	Positive (NM Serogroup B)	No	Positive (NM Serogroup B)	Positive (NM Serogroup B)	Positive (NM Serogroup B)
Died	Yes	Yes	Yes	No	No	No

ICU=Intensive Care Unit; NM=*Neisseria meningitidis*; PCR=Polymerase chain reaction

Transportation and funeral costs were not considered because these were not available. Vaccination costs were not included as no vaccine was available at the time. All costs are expressed in US dollar (2011) as cost per 1,000 inhabitants.

## RESULTS

### Patients and contacts

During the period of the outbreak (from 21 February to 21 March 2012), we identified 6 patients and 46 contacts (family and childcare). The suspected cases were detected from CH, and they were reported to the DADIS. Both institutions ran an active surveillance network in the neighbourhood cases, which consisted of two phases: one was the control of the outbreak, and the other was the monitoring of diseases/cases.

**Figure 1.**
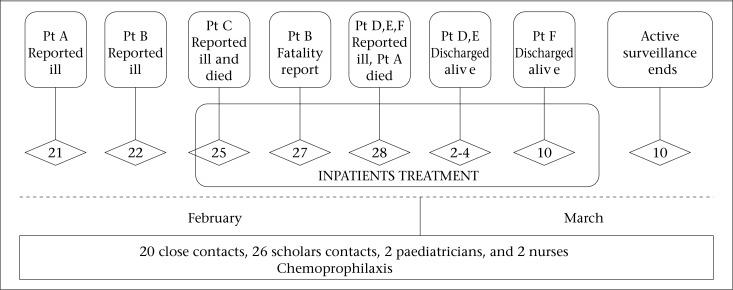
Timeline of major events involving invasive meningococcal disease outbreak in a Colombian Caribbean City, and response of DADIS and CH Cartagena, 21 February–21 March 2012

Patient, clinic, and laboratory characteristics involving confirmed, probable and suspect cases of meningococcal disease (N=6) are shown in Table 1. The timeline of the major events involving the invasive meningococcal disease outbreak in Cartagena de Indias is described in Figure 1.

On 28 February at approximately 3:20 pm, CH notified that patient A had died. This patient was a cousin of patient B and C, both of whom died on 27 and 25 February respectively. Patient A had an isolated *N. meningitidis* from blood cultures, and pathologic examination showed that this was compatible with meningococcaemia and meningitis. On 28 February and 2 March, three additional patients had been hospitalized with fever and abdominal pain (patient D, E, F). These three illnesses were eventually found to be cases of *N. meningitidis* infection, based on their positive blood culture results. For this reason, the DADIS field team expanded chemoprophylaxis (Figure 1) eligibility to select direct contact with the patients who lived more than 3 blocks away and had been in contact with them 2 weeks before the outbreak.

**Figure 2.**
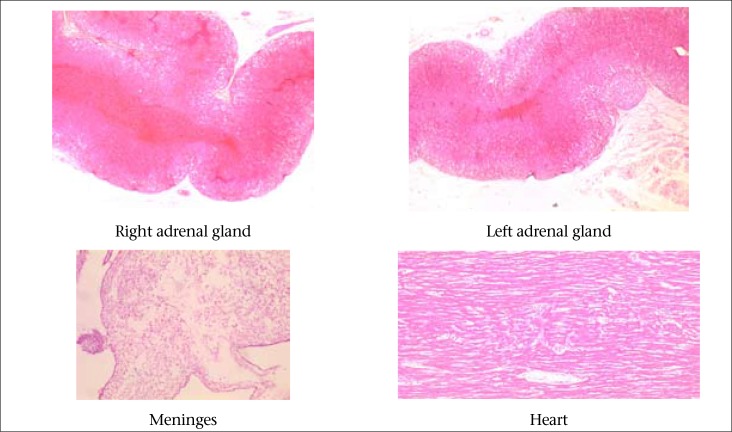
Pathologic findings compatible with Friderichsen Waterhouse Syndrome: Patient C

Once *N. meningitidis* was confirmed, DADIS and CH conducted a routine contact investigation. Since the recent acquisition of *N. meningitidis* is the risk factor to develop in a systemic infection, the DADIS and CH established preventive measures, such as chemoprophylaxis with ceftriaxone to contacts identified, hygiene measures and education in the neighbourhood of the index case. The DADIS and CH found that a 5-year old patient (case C) had died in an unexpected way, with signs and symptoms consistent with involvement of the central nervous system and systemic symptoms in a primary-care centre located in the same neighbourhood where the index case (patient A) and patient B resided, and close to the CH. Case C tested positive for NM by culture, and specimens autopsy confirmed that this patient also had the Waterhouse Friderichsen Syndrome (Figure 2). All three patients were cousins, slept in the same house and in the same bed. The average age of patients was 4.6 years, and the case-fatality rate was 50%.

The occurrence of three cases within a 48-hour period prompted DADIS and CH to begin outbreak control measures. This included the provision of chemoprophylaxis to children and other patient contacts to provide short-term protection of the population at risk. DADIS and CH mobilized personnel to operate an interim mass chemoprophylaxis at the neighbourhood and the CH where these cases were identified. In addition, many activities were conducted, such as a population census in the neighbourhood of the index case, identification of the close contacts to the index case, and clean-up and organization of the outbreak area to reduce overcrowding a little (15,26). Chemoprophylaxis was administered for a period of 2 weeks (27 February through 8 March). The DADIS field epidemiology team also administered antibiotic therapy to the population at risk.

Among 20 close contacts that were admitted to hospital (CH) for medical check-up, two (cases D and E) were found with symptoms of fever, for which they were hospitalized, and blood cultures were taken that confirmed NM serogroup B. These two cases received antibiotics treatment and were discharged from the hospital satisfactorily; they were followed for three weeks without any relapse or complication.

### Chemoprophylaxis and vaccination

On 2 March, the Health National Institute (HNI) of Colombia reported to the DADIS and CH that isolation was a serogroup B *Neisseria* in all patients. Immediately, the local health department (DADIS and CH) disseminated the information about the outbreak of meningitis to the community through local and national newspapers to alert them about the situation and allow the authorities to take necessary steps to contain the outbreak.

Intramuscular ceftriaxone was chosen as the agent for children and adults among at-risk population to ensure rapid initiation of chemoprophylaxis (7,27).

During the response phase (investigation and outbreak management from 27 February to 8 March), 50 doses of ceftriaxone were administered as prophylaxis. DADIS did not recommend meningococcal vaccination as a meningococcal vaccine was not available in Colombia at the time of outbreak.

To control the outbreak, a chemoprophylaxis clinic was set up in the population at risk. We applied 50 doses of ceftriaxone initially to the 20 close contacts of the index case (patient A) (19-22). The close contacts were given chemoprophylaxis within the first week of identification of the index case. Between the first and second week after the index case was identified, 26 ceftriaxone doses were applied to 26 children identified as contacts of the school where index case patient was studied. None of these contacts was identified as symptomatic at school (22). Finally, prophylaxis with ceftriaxone was given to 2 paediatricians and 2 nurses in the CH who provided care to the index case (patient A) and were in contact with the secretions of this patient. The whole population was kept under surveillance for three weeks following the outbreak. No new cases were confirmed during this period. The average age of the children who received prophylaxis was 5.4 years (95% CI 4.2-6.8).

### Total cost

The 6 cases were associated with a total cost of US$ 735 (or US$ 0.80 per 1,000) (Table 2) for the disease response phase of the outbreak. This cost corresponds to the cost associated with the control of the outbreak and includes personnel costs, and costs of office supplies and chemoprophylaxis. This may be underestimated because the transportation and funeral costs were not considered in this estimate. The main contributors of the costs of disease response phase were personnel costs, and cost of chemoprophylaxis representing 68% and 32% of the total cost respectively.

The total costs of monitoring the disease cases (or disease surveillance costs) were estimated at US$ 3,935 and were attributed mainly to personnel costs. Costs associated with a vaccination programme were not included in the calculations of the outbreak because the Cuban vaccine strategy (the one that would have been optimal for controlling the outbreak in this setting) was not available, and the serogroup of NM isolated in the outbreak was serogroup B, which is not currently available for a vaccine with long-term protection.

In addition to the costs associated with the disease response phase and the disease surveillance phase, there were costs relating to hospital care provided during the meningococcal outbreak. The total cost of hospital care of patients during an outbreak was US$ 4,921, contributing 67.6% of the total cost for patient A and B because these patients were admitted to ICU (Table 3).

**Table 2.** Cost of an outbreak in Cartagena de Indias, Colombia (US$ as of 2011)

Resource	Disease response phase (Control of the outbreak)	Disease surveillance phase (Monitoring of disease cases)
Personnel	US$ 502.3	US$ 3,921.9
Office supplies	NR	US$ 13.18
Chemoprophylaxis	US$ 232.8	Not applied
Meningococcal C conjugate vaccine	Not applied	Not applied
Meningococcal polysaccharide vaccine	Not applied	Not applied
Total cost	US$ 735.1	US$ 3,935.08
Cost per 1,000 inhabitants	US$ 0.8	US$ 4.1

NR=Not reported

**Table 3.** Hospital care costs of cases of meningococcal outbreak

Patient	Drugs (US$)	Test (US$)	Procedures (US$)	Regular ward LOS (US$)	ICU LOS (US$)	Total (US$)
Patient B	246.4	1,010.6	16.8	-	913.7	2,187.5
Patient A	155.5	235.0	293.3	-	456.8	1,140.6
Patient D	19.0	123.2	16.8	303.7	-	462.8
Patient F	40.7	156.6	-	607.4	-	804.8
Patient E	16.5	140.6	16.8	151.9	-	325.7
Total	478.2	1,666.0	343.7	1,063.0	1,370.5	4,921.4
Median	40.7	156.6	16.8	151.9	-	804.8
Mean	95.6	333.2	68.7	212.6	274.1	984.3
Cost per 1,000 inhabitants	0.5	1.7	0.4	1.1	1.4	5.1

LOS=Length of stay

## DISCUSSION

This is the first report of an outbreak of NM serogroup B in Colombia where we made a detailed description of detection, intervention, control, prevention, monitoring, cost of cases and surveillance. The paper emphasizes early intervention by the local health services (DADIS and CH), timely application of chemoprophylaxis, and public-health measures at the district and school levels. The paper also highlights the different diagnostic methods for identification of the disease—clinical, microbiological, molecular, and pathologic—and estimating the direct cost of care of case and the outbreak.

Annual incidence of invasive meningococcal infection is approximately 0.5 cases per 100,000 populations (3,28). Incidence rates for serogroup B vary between 3 and 50 per 100,000 population during epidemics and between 0.4 and 4 per 100,000 for endemic areas (CDC) (6,29).

Serogroup B causes over 50% of cases in infants younger than 1 year of age. However, during an outbreak, the proportion of cases was higher in older children (7,30). The incidence rate (0.26 to 0.63×100,000 inhabitants), the case-fatality rate (50%), and displacement of the infection to older age-groups (average 4.6 years, SD 3.5) were consistent with those reported in the literature to be predictive indicators of outbreaks or epidemics (31-33). Case-fatality rates were higher for outbreak-associated cases than for sporadic cases (34). The fatality rate of meningococcal disease in Colombia is reported between 19% and 37% (6). In this outbreak, given the rapid onset of cases, health institutions (DADIS and CH) had to intervene quickly.

A large outbreak caused by *N. meningitidis* serogroup B strain (NZ98/254) reported in the literature is that from New Zealand, which estimated a peak incidence of 17.4/100,000 (35,36). In the Americas, some countries have experienced significant epidemics of *N. meningitidis* serogroup B: reported for its size and lethality of epidemics in Cuba (37), Brazil (38,39), United States, particularly in the state of Oregon (40), and Dominican Republic (41).

In Colombia, until 1993, information on cases of meningococcal disease was collected monthly, using the form SIS-12 (mandatory reportable disease). In recent years, with the establishment of decree 3518 of October 9 in 2006, the Ministry of Social Protection was created. This Ministry was established to regulate the system of public health surveillance focused on bacterial meningitis, especially those caused by *Neisseria meningitidis, Haemophilus influenzae*, and *Streptococcus pneumoniae.* It was also established to improve the structure of the Action Alert System (AAS) by the Ministry of Health and enhance the surveillance system of various diseases. Based on the above, Colombia has reported the following incidence rates of meningococcal disease: rates from 0.55 to 1.02 per 100,000 in different years from 1994 until 2011 (6). In Colombia, an outbreak of 5 cases was reported in 1998 in a city on the border with Brazil, the incidence was estimated at 14 per 100,000 with a mortality of 40% (6).

The primary means of preventing the spread of meningococcal disease is antimicrobial chemoprophylaxis. In this outbreak, chemoprophylaxis was implemented as the definitive outbreak control measure based on three reasons: (i) the prompt onset of cases, given that close household contacts of patients are at 500- to 1,000-fold increased risk for the disease (42); (ii) the serogroup of *N. meningitidis* isolated in the outbreak was serogroup B, which is not currently available for a vaccine with long-term protection; (iii) situations in which mass chemoprophylaxis could be successful, including those involving limited or closed populations, such as a single school or residential facility. This is especially important in serogroup B outbreaks since vaccines cannot be used for control and prevention (7).

The costs in the control phase of the outbreak included the cost of staff and chemoprophylaxis used. The costs in the monitoring phase were provided by personnel costs. This is in contrast with the costs in the control of an outbreak reported in Vila Bardina, Campinas (Brazil) in 2011, which reported 3 cases with a total cost of managing outbreak at US$ 34,425. The largest contributor of the cost in the Campinas outbreak was the use of vaccines against meningococcus (a polysaccharide and a serogroup C conjugate) and personnel costs, which was not used in the current outbreak because no conjugate vaccine is currently available for serogroup B.

Conventional polymerase chain reaction (PCR) was performed on two CSF samples of patient A and B, determining the *crg*A gene. However, the results of the PCR were negative (43). The National Institute of Health of Colombia performed real-time PCR in the same CSF samples of patient A and B. In these two samples (CSF), the result of the real-time PCR was positive for *N. meningitidis* serogroup B. Therefore, we can conclude that these invasive strains of *N. meningitidis* isolated from patients in the outbreak, carry the cps operon, which contains within its genes regulating gene expression *ctr*A—the capsule—and not the gene *crg*A, a gene expression of adhesion (44,45).

### Strengths and limitations

The strength of the outbreak investigation was the early approach, coordinated and effective response by health authorities as the DADIS and CH and a mass chemoprophylaxis that was implemented, which helped control the outbreak. We emphasize the use of different diagnostic methods to find the aetiology of the cases: culture specimen, necropsy, and polymerase chain reaction.

One of the limitations of this research was that the microcosting analysis was not done with all the cases. We had to exclude patient C because no data were available on this patient. Another limitation was the timeframe considered for the analysis. We conducted a cost analysis during the outbreak and 3 weeks following the outbreak. The unavailability of a polysaccharide vaccine against serogroup B used, especially in serogroup B outbreaks, was another limitation (46) as well as the costs associated with education and dissemination of information that were not included. The above may underestimate the total costs of the outbreak. The meningococcal B strains were not subjected to Multilocus Sequence Typing (MLST) because this is not routinely done; so, we did not know which serotype or serosubtype was circulating.

### Conclusions

Meningococcal disease outbreaks generate rapid, intensive public-health response because it is a fatal disease. The meningococcal serogroup B was the cause of the outbreak that is described in this paper. A timely identification and management was critical for the control of outbreak. A mass chemoprophylaxis was implemented. The costs excluded from the analysis (costs associated with patient C, transportations and funeral cost, serotype/serosubtype B vaccination costs) resulted in an underestimation of the total costs of the outbreak.

### Recommendations

Strengthening the surveillance systems, including research on the prevalence of serotype/serosubtype B, is recommended. Cost and impact of a community outbreak of meningococcal disease incurs considerable costs to the society.
